# Multiple-patterning colloidal lithography-implemented scalable manufacturing of heat-tolerant titanium nitride broadband absorbers in the visible to near-infrared

**DOI:** 10.1038/s41378-020-00237-8

**Published:** 2021-03-02

**Authors:** Dasol Lee, Myeongcheol Go, Minkyung Kim, Junho Jang, Chungryong Choi, Jin Kon Kim, Junsuk Rho

**Affiliations:** 1 Department of Mechanical Engineering, Pohang University of Science and Technology (POSTECH), Pohang, 37673 Republic of Korea; 2 Department of Chemical Engineering, Pohang University of Science and Technology (POSTECH), Pohang, 37673 Republic of Korea; 3 National Institute of Nanomaterials Technology (NINT), Pohang, 37673 Republic of Korea

**Keywords:** Nanophotonics and plasmonics, Structural properties

## Abstract

Broadband perfect absorbers have been intensively researched for decades because of their near-perfect absorption optical property that can be applied to diverse applications. Unfortunately, achieving large-scale and heat-tolerant absorbers has been remained challenging work because of costly and time-consuming lithography methods and thermolability of materials, respectively. Here, we demonstrate a thermally robust titanium nitride broadband absorber with >95% absorption efficiency in the visible and near-infrared region (400–900 nm). A relatively large-scale (2.5 cm × 2.5 cm) absorber device is fabricated by using a fabrication technique of multiple-patterning colloidal lithography. The optical properties of the absorber are still maintained even after heating at the temperatures >600 ^∘^C. Such a large-scale, heat-tolerant, and broadband near-perfect absorber will provide further useful applications in solar thermophotovoltaics, stealth, and absorption controlling in high-temperature conditions.

## Introduction

Broadband perfect absorbers^1–8^ have many possible uses, such as thermophotovoltaics^9–11^ and thermal emitters^12–14^, but practical applications have been limited by the lack of scalable fabrication method and by their poor thermal durability. Broadband perfect absorbers are typically realized by plasmonic nanostructures bonded to metallic reflective layer separated by a dielectric spacer in which noble metals are commonly used^1,15^, but these materials are costly. Therefore, refractory materials have been introduced as candidates for broadband absorbers due to their high-temperature tolerance, chemical stability, mechanical durability, and low cost^16–20^.

Titanium nitride (TiN) has comparable plasmonic properties to gold (Au) in the visible and near-infrared (NIR) region, and has therefore been used in solar heat generators and photodetectors^21–24^. However, most previous studies have used electron beam lithography to pattern the nanostructures, and this method limits a scale to a few hundred micrometers. To be implemented in practical applications, a method to fabricate TiN nanostructure in a large scale is in high demand.

Colloidal lithography is widely used to create periodic 2D nano-sized patterns on a variety of substrates. It can fabricate templates or masks for use in creation of various nanostructures, such as nanohole, nanodisk, nanopillar, and nanocone, which have been applied to various photonic devices^25–28^. A consecutive use of the colloidal lithography, which is referred to as multiple-patterning colloidal lithography (MPCL), enables fabrication of hierarchical nanostructures, such as hollow nanocone, nanotower, and nanoring^29–34^. However, these methods have only been used to etch silicon or polymers, while not being extended to etch other materials.

Here, we use MPCL to fabricate a large-scale (2.5 cm × 2.5 cm) near-perfect absorber that is composed of ring-shaped TiN structures. To best of our knowledge, it is the first demonstration of applying MPCL to etch a refractory metal, which is focused on the fabrication approach to overcome the limitations of practical use as a large-scale absorber. The device has polarization-independent absorption of 95.4% under normal incidence and high absorption at incident angles up to *θ* = 40^∘^ at visible and NIR wavelength (400 < *λ* < 900 nm). The near-perfect absorption of our device remains even after heating at temperature up to 600 ^∘^C. We expect that our cost-effective and scalable absorber is a promising candidate for large-scale applications, such as photothermal devices and thermal emitters that require polarization-independent, angle-insensitive, heat-tolerant, and broadband absorption.

## Results and discussion

### Device optimization and fabrication

The proposed broadband ring-shaped TiN absorber has a metal–insulator–metal (MIM) structure that uses a perfectly-reflective TiN layer, silicon dioxide (SiO_2_) dielectric layer, and a top composed of ring-shaped TiN nanostructures in a hexagonal array. The ring-shaped nanostructure has advantages in absorption due to its better impedance matching with air compared to disk-shaped structures^16,19^, and can be fabricated in large scale using MPCL.

**Fig. 1:**
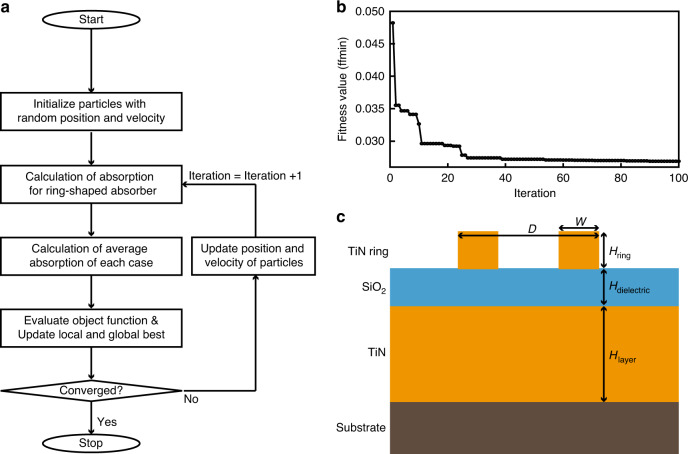
Optimization and schematic of a ring-shaped TiN absorber. **a** Flow chart of the particle swarm optimization (PSO) algorithm to optimize the broadband titanium nitride (TiN) ring-shaped perfect absorber. **b** Fitness curve of PSO algorithm. The fitness value converges to 0.026 after 100 iterations. **c** Schematic and parameters of a ring-shaped TiN absorber. The optimized dimensions are *P* = 300 nm, *D* = 181.09 nm, *W* = 79.27 nm, and *H*_ring_ = 60 nm, *H*_layer_ = 150 nm, and *H*_dielectric_ = 58.09 nm

Particle swarm optimization (PSO)^35^ is used to optimize the structural parameters of the TiN absorber to have the highest absorption in the visible and NIR region. For this purpose, we develop a lab-built PSO by linking commercial software (COMSOL Multiphysics 5.5, Livelink for MATLAB, MATLAB 2019; Fig. 1a). First, finite-element method simulation is performed to calculate the absorption from randomly selected initial parameters. The objective function indicates the function to be optimized, and is defined by subtracting the square of the average absorption in 400–900 nm region from unity. Then the optimization proceeds to minimize the fitness value of this objective function. At each iteration, minimum fitness value is set as the local best. If the new local best is smaller than the minimum fitness value in history (global best), the local best is set as the new global best. The cycle is repeated until fitness converges or until the maximum number of iterations is applied.

The outer diameter (*D*) and width (*W*) of the ring structure of the top ring-shaped nanostructure, and the thickness (*H*_dielectric_) of the SiO_2_ dielectric layer are considered as parameters for optimization, and other parameters such as period (*P*) and thickness of ring structure (*H*_ring_) are fixed due to the feasible fabrication for a TiN absorber. The initial ranges of parameters are set to 0 < *D* < 300 nm and 10 < *W* < 150 nm to prevent them from exceeding the outer diameter. One hundred iterations are repeated until the optimal value is found. Each iteration considers 30 particles, so a total of 3000 particles are evaluated to identify the final parameters for optimal ring-shaped absorber. After 100 iterations, the fitness converges to <0.026 (Fig. 1b).

The optimal dimensions of the proposed ring-shaped TiN absorber (Fig. 1c) are *P* = 300 nm in a hexagonal array, *D* = 181.09 nm, *W* = 79.27 nm, and *H*_ring_ = 60 nm on the layers deposited by TiN (*H*_layer_ = 150 nm) and SiO_2_ (*H*_dielectric_ = 58.09 nm) in sequence. As such, optimization techniques can help in designing optical structures and can be further advanced using machine learning or deep learning^36–40^.

**Fig. 2:**
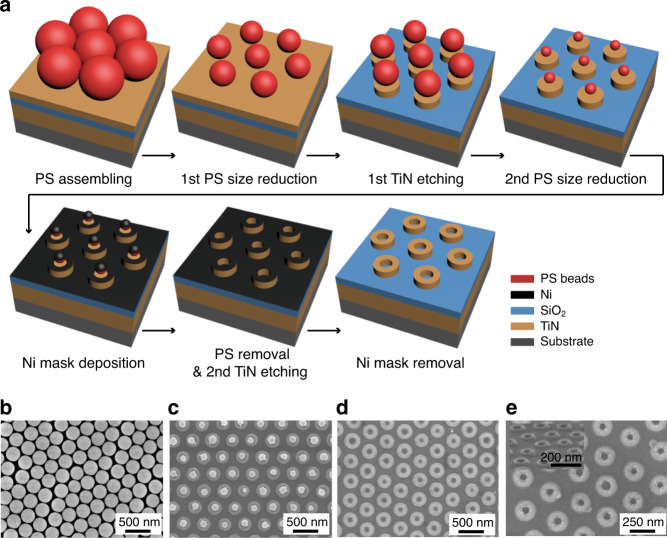
Fabrication of a ring-shaped TiN absorber. Hexagonally patterned TiN absorber with multiple-patterning colloidal lithography (MPCL). **a** Schematic illustration of the process. Highly ordered TiN ring-shaped structures are fabricated using MPCL. Scanning electron microscopy images **b** of hexagonally packed PS nanospheres monolayer, **c** after second PS size reduction, **d** after PS removal, **e** of final ring-shaped TiN absorber. Inset shows the tilted view of final ring-shaped TiN absorber

Schematic illustration to fabricate the TiN ring-shaped absorber is described (Fig. 2a). TiN–SiO_2_–TiN MIM structure is prepared on silicon substrate, then MPCL is used to prepare highly ordered TiN ring-shaped structures; the period and diameters are determined by controlling the size of the polystyrene (PS) nanosphere and the oxygen plasma etching time. A PS monolayer is firstly prepared on the top layer of the prepared MIM structure (Fig. 2b). PS nanospheres with a diameter of 300 nm are chosen to match the period between nanoring structures. The hexagonally arranged PS monolayer is etched using oxygen plasma to reduce the PS diameter to the optimized *D*. A top TiN layer is etched using reactive ion etching (RIE) with Cl_2_ and BCl_3_ gas by using the PS beads as a mask. The RIE yields a nanodisk structure, then a second PS etching is performed to achieve the optimized *W* (Fig. 2c). A 8-nm thick nickel (Ni) layer is deposited as a hard mask under Cl_2_ gas using electron beam (e-beam) evaporator; then all remaining PS nanospheres are removed by sonication in toluene for an hour. The second TiN etching is performed (Fig. 2d). The Ni mask is removed by 1 mol/L HCl solution to complete the final TiN nanoring structure. These processes yields ring-shaped nanostructure array that is consistent with the optimized parameters (Fig. 2e). In order to fabricate elaborate nanostructure, there are several issues to be considered. First, the etching conditions must be precisely controlled^41^ because the intrinsic property of dry etching process can make the ring-shaped structure slightly tapered. Second, the uniformity of the structure can be improved by using nanospheres with lower size distribution. Third, random tilting of the etched PS nanospheres during the RIE etching can be prevented by enhanging the adhesion between PS nanospheres and TiN layer by heating^31^.

### Optical properties

**Fig. 3:**
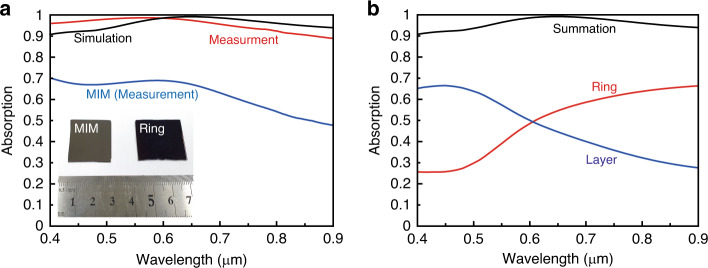
Absorption spectrum and the total power dissipation density. **a** Simulated (black line) and measured (red line) absorption spectra of ring-shaped absorber and measured absorption spectrum of metal–insulator–metal (MIM) structure (blue line) for comparison. Inset: photographs of fabricated absorbers. **b** The total power dissipation density of top ring-shaped TiN nanostructure array (red line), bottom TiN layer (blue line) to examine absorption contribution of two TiN layers. The summation of two dissipation densities (black line) is equal to the simulated absorption spectrum

For numerical demonstration, we calculate absorption of the ring-shaped TiN absorber; refractive indices of TiN and SiO_2_ are taken from the literature^42–44^. The result shows 95.9% average absorption at 400–900 nm and the highest absorption at 650 nm (Fig. 3a). High absorption of the proposed design is experimentally demonstrated using spectroscopy. Unpolarized light is illuminated at normal incidence onto the sample. The measured spectra of ring-shaped absorber and MIM structure (Fig. 3a) show an average absorption of 95.4%. Compared to the MIM layer without ring-shaped structure, it absorbs >31% light in the visible–NIR region. The slight difference between measurement and simulation is attributed to fabrication imperfections of placement and size distributions of the nanostructure. Photographs (Fig. 3a, inset) of ring-shaped absorber and MIM structure show obvious darkness obtained by the absorption characteristic.

To understand the mechanism of the high absorption, we examine the total power dissipation density

1 $${Q}_{\mathrm{abs}}=< {J} \cdot {E}>=\rho {\left|E\right|}^{2}=\frac{1}{2}{\epsilon }_{0}\epsilon ^{\prime\prime}_{r}\omega {\left|E\right|}^{2}$$

where *ρ* is the electrical conductivity, *ϵ*_0_ is the permittivity of a vacuum, $\epsilon ^{\prime\prime}_{r}$ is the imaginary part of the relative permittivity of the material, *ω* is angular frequency, and $\left|E\right|$ is the amplitude of the total electric field inside the material. *Q*_abs_ is extracted from each TiN layer. *Q*_abs_ can be a criterion to evaluate the contribution of the absorbed electromagnetic power from each TiN layer.

Absorption can be explained as a field localization by localized surface plasmons (LSPs) and by intrinsic loss of the material. The absorption contributions of each TiN layer verify the specific mechanisms (Fig. 3b). The LSPs occur at the surface near the nanostructures, so the absorption by the LSP should be caused by the ring-shaped nanostructure. In the region of *λ* > 600 nm, TiN exhibits metallic properties, which lead to plasmonic resonance. Thus, the absorption is dominated by the ring structure. However, compared to noble metals, such as Au, TiN has weak metallic properties, so it has localized fields inside the ring structure. In addition, unlocalized waves can pass through the top layer and arrive at the underlying TiN bulk layer due to its large skin depth. The broad high absorption is attributed to two absorptions in the ring structure and bulk TiN layers simultaneously. In the range of *λ* < 600 nm, the bottom TiN layer contributes more absorption than the ring structure does. This difference means that the intrinsic loss at the bulk TiN layer is dominant. TiN can be a lossy dielectric material in this region, so the bottom TiN layer absorbs a large portion of incident light at high frequencies.

**Fig. 4:**
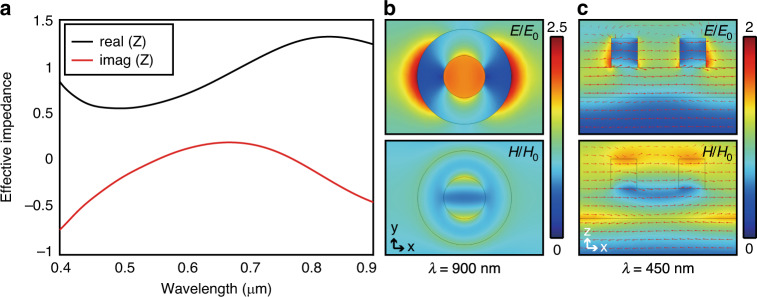
Retrieved effective impedance and field distributions of a ring-shaped TiN absorber. **a** Effective impedance of a ring-shaped TiN absorber retrieved from S-parameters. **b** Simulated electric and magnetic field distribution at 900 nm along *xy*-plane. **c** Simulated electric and magnetic field distribution at 450 nm along *xz*-plane with current density (red arrow)

For further investigation, the impedance-matching condition of the proposed absorber is analyzed. Under the assumptions that the absorber structures are homogeneous, the effective impedance (Fig. 4a) of the proposed structure is retrieved using the S-parameter retrieval method as^45^

2 $$z=\pm \sqrt{\frac{{(1+{S}_{\text{11}})}^{2}-{S}_{\,\text{21}\,}^{2}}{{(1-{S}_{\text{11}})}^{2}-{S}_{\,\text{21}\,}^{2}}}$$

The effective impedance is calculated under the condition of plane wave from air. The effective impedance shows the real part of the calculated effective impedance is close to 1, and the imaginary part is close to 0, so the impedance matches that of free space in the visible and NIR regions. Thus, reflection is weak and absorption is dominant over the desired wavelength region.

Normalized plots of electric and magnetic field distribution on the *xy*- and *xz*-planes are plotted in Fig. 4b, c. We analyzes the fields at *λ* = 900 nm and *λ* = 450 nm, which are the resonance peaks in TiN ring and layer, respectively (Fig. 3b). At *λ* = 900 nm, the electric field distribution shows an electric dipole resonance at the top ring antenna, whereas the magnetic field distribution is relatively weak. This result demonstrates that the absorption is mainly caused by the ring-shaped nanostructure. In contrast, at *λ* = 450 nm, the magnetic field distribution shows a magnetic dipole resonance as a result of coupling between the TiN layers. The antiparallel current density *J*_*d*_ leads a loop bringing an artificial magnetic dipole moment. Thus, the ring-shaped TiN absorber excites electric and magnetic resonances, which increase the localized electromagnetic field at the corresponding wavelength.

**Fig. 5:**
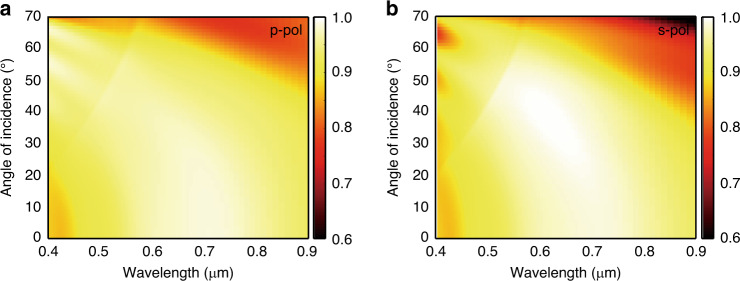
Simulated absorption spectra of the TiN absorber with an angle of incidence. Color maps represent absorption for both **a** p- and **b** s-polarization light, respectively. High absorption >90% is obtained at an angle of 40^∘^ for both polarizations

The ring-shaped absorber is radially symmetrical, so its absorption is not greatly affected by polarization or incident angle of light at *θ* < 40^∘^ (Fig. 5a, b). However, at *θ* > 40^∘^, absorption decreases because the confinement of the EM field weakens. This degradation occurs because the resonances are sensitive to polarization and incident angle. Nevertheless, our observation confirms that absorption >90% is maintained at an angle of 40^∘^ in both p- and s-polarizations. This characteristic means that the proposed absorber can be widely used in practical fields, such as photothermal applications.

### Heat tolerance

**Fig. 6:**
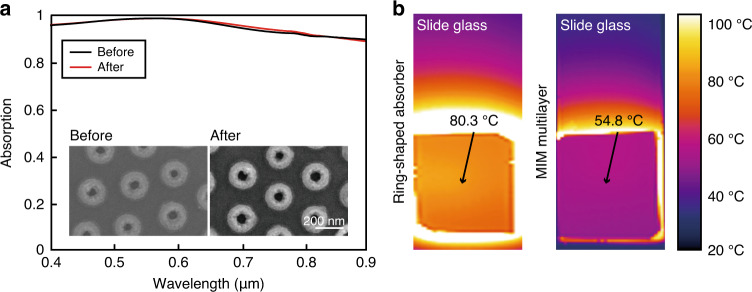
Heat tolerance of a ring-shaped TiN absorber. **a** Absorption spectra of the TiN absorber before (black line) and after (red line) annealing at the temperature of 600 ^∘^C, and SEM images. **b** Photographs captured by an infrared camera, of (left) ring-shaped TiN absorber and (right) MIM structure. The fabricated absorbers are attached to slide glass and illuminated using a xenon lamp. Temperature of the TiN absorber increases over 80 ^∘^C due to its near-perfect absorption, whereas MIM structure has much lower temperature

The proposed absorber is expected to have a great heat tolerance as a result of TiN’s high melting point of 2930 ^∘^C. To confirm the heat tolerance of the ring-shaped TiN absorber, it is heated in a vacuum chamber at 600 ^∘^C for 6 h and cooled down to room temperature. We confirm that the TiN absorber maintains its shape and optical properties even after heating at high temperature (Fig. 6a). Even at a high temperature near 600 ^∘^C, TiN and SiO_2_ show only slight changes in their permittivities, but each retains its original optical properties quite well^46,47^. If the TiN absorber is protected by an atomic layer deposited coating or sealing, it may tolerate even much higher temperatures^16^.

For practical applications such as solar and photovoltaic fields, we test the photothermal stability of the TiN absorber under natural-like condition in an open environment. White light from a high-power xenon lamp is collimated and illuminated at fabricated samples that are fixed on slide glass. The sample is far enough from the source to avoid a direct thermal effect by the high temperature of the lamp. MIM structure is measured and compared to the TiN absorber for the photothermal activity. After being illuminated for same duration (5 min), the temperature of each sample is captured using an infrared camera (Testo 885, Testo; Fig. 6b). The temperature of the TiN absorber increases to >80 ^∘^C, which is 25.5 ^∘^C higher than that of MIM structure. The high absorption in our devices, combined with the ring-shaped nanostructure opens a gateway for efficient energy extraction in photothermal applications, due to advantages of low-cost and compatibility with large-scale fabrication.

## Conclusion

We have reported a large-scale heat-tolerant TiN broadband absorber that shows >95% of unpolarized light absorption in the visible–NIR range of 400 < *λ* < 900 nm. We propose the implementation of cost-effective MPCL with refractory metal for fabrication of a centimeter scale ring-shaped absorber. Because of the heat-tolerant characteristic, the TiN absorber retains high absorption after heating up to 600 ^∘^C. This absorber will find wide applications in solar thermophotovoltaics, stealth, and further absorption controlling in high-temperature conditions.

## Materials and methods

### Materials

Suspensions of PS nanospheres with 0.3-μm diameter were used (Thermo Fisher Scientific). Absolute ethanol and toluene were used (Sigma Aldrich). Deionized water was obtained from a water purification system (Ultima Duo 300, Azzota Scientific). Hydrochloric acid (1 mol/L) was used (Samchun Chemical).

### Fabrication

TiN and SiO_2_ layers of MIM were deposited using DC Sputtering and e-beam evaporator. The PS colloidal monolayer were self-assembled into hexagonally closed-packed arrays on the TiN layer by using an air interface method. The diameters of the PS nanospheres were reduced by oxygen RIE (VITA, Femto Science) at 80 W with 30 sccm O_2_. The top TiN layer was first etched at 100 W with 25 sccm Cl_2_ and 100 sccm BCl_3_. Then a second PS etching was performed in the same conditions. Then an 8-nm thick Ni layer was deposited by e-beam evaporator at a deposition rate of 0.3 Å/s. Then, the PS nanospheres were removed by sonication in toluene. A second TiN etching was performed in the same condition as the first etching. Finally, the Ni mask was easily removed by immersion in 1 mol/L hydrochloric acid for 10 min, followed by rinsing with DI water.

### Characterization

A UV–VIS–NIR spectrometer (Cary 5000, Varian Co.) with diffuse reflectance accessory (Internal DRA-2055, Varian Co.) was used to characterize the absorption of the large-area sample in the wavelength range 400–900 nm, using white reflectance standards (Ocean optics) as a reference. The wavelength-sampling step was 1 nm. We defined absorption as 1-reflectance.

Fabricated TiN absorber structures were observed using a scanning electron microscope (Hitachi S4800) at 3 kV.

The thermal images were taken by an infrared camera (Testo 885, Testo), and the temperature was determined using Testo IRsoft software. The emissivity of 0.34 and room temperature of 21 ^∘^C were used for calibration. For calibration, we used an approach to directly measure the emissivity value of the infrared camera using a contact thermometer.
